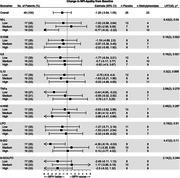# Predictive and monitoring value of blood‐based biomarkers for apathy treatment in Alzheimer’s disease

**DOI:** 10.1002/alz.095596

**Published:** 2025-01-09

**Authors:** Krista L Lanctôt, Shankar Tumati, Danielle Vieira, Kritleen Kaur Bawa, Ana C. Andreazza, Roberta Scherer, Paul B. Rosenberg, Christopher H. van Dyck, Prasad R Padala, Olga Brawman‐Mintzer, Anton P. Porsteinsson, Alan J. Lerner, Suzanne Craft, Allan I. Levey, William J Burke, Jamie Perin, David Shade, Jacobo Mintzer, Nathan Herrmann

**Affiliations:** ^1^ Sunnybrook Research Institute, Toronto, ON Canada; ^2^ University of Toronto, Toronto, ON Canada; ^3^ Neuropsychopharmacology Research Group, Sunnybrook Research Institute, Toronto, ON Canada; ^4^ Johns Hopkins University, Baltimore, MD USA; ^5^ Johns Hopkins University School of Medicine, Baltimore, MD USA; ^6^ Alzheimer’s Disease Research Unit, Yale School of Medicine, New Haven, CT USA; ^7^ Central Arkansas Veterans Healthcare System, North Little Rock, AR USA; ^8^ Medical University of South Carolina, Charleston, SC USA; ^9^ University of Rochester School of Medicine and Dentistry, Rochester, NY USA; ^10^ Department of Neurology, University Hospitals Cleveland Medical Center, Cleveland, OH USA; ^11^ Wake Forest University School of Medicine, Winston‐Salem, NC USA; ^12^ Emory University, Atlanta, GA USA; ^13^ Banner Alzheimer’s Institute, Phoenix, AZ USA; ^14^ Johns Hopkins, Baltimore, MD USA

## Abstract

**Background:**

Apathy in Alzheimer’s disease improves with methylphenidate (MPH) but treatment response was found to vary depending on clinical factors. Here, we explored whether underlying biological factors assessed by blood‐based biomarkers of neurodegeneration, inflammation and oxidative stress affect apathy treatment response.

**Method:**

A subset of participants from the Apathy in Dementia Methylphenidate Trial 2 (ADMET 2) were included in this study whose blood samples were available at baseline and at the 6‐month treatment completion. Apathy was assessed with the Neuropsychiatric Inventory apathy subscale (NPI‐A, range: 0‐12). Blood concentrations of (i) neuronal damage: neurofilament light (NFL) and S‐100B (available at baseline only), (ii) inflammation: interleukin (IL)‐6, IL‐10, Tumor Necrosis Factor‐alpha (TNFα), and (iii) oxidative stress: lipid hydroperoxide (LPH), 4‐hydroxynonenal (4‐HNE), 8‐isoprostane (8‐ISO) were obtained with ELISA assays. Biomarkers were normalized by log transformation and pareto scaling. Predictive value of biomarkers was assessed by examining differences in treatment response between each biomarker tertile level. We also assessed whether biomarkers improved predictive models for established clinical predictors. Monitoring value was assessed by linear mixed models with NPI‐A as the dependent variable and interaction between biomarker and time or biomarker and treatment as the independent variable.

**Result:**

Among 55 participants (MPH: 24, age: 75.4 years [standard deviation (SD): 7.6], MMSE: 19.9 [SD: 4.9]) at baseline and 49 at the 6 month end point, the change in NPI‐A showed a greater than 2‐point difference between tertiles of NFL (6.8 points), TNFα (4.2 points) and 8‐ISO (5.5 points) (Fig. 1). Treatment response prediction improved by adding NFL with cholinesterase use (likelihood ratio test[lrt]: 4.6, p: 0.03), presence of agitation (lrt: 4.4, p: 0.04) or presence of anxiety (lrt: 4.5, p: 0.04); no added value was found with TNFα and 8‐ISO. As a monitoring biomarker, TNFα (but not NFL and 8‐ISO) levels over time were associated with NPI‐A score (t: 2.69, p: 0.009).

**Conclusion:**

Blood‐based biomarkers of neurodegeneration, inflammation and oxidative stress are associated with apathy and affect treatment response, indicating potential predictive and monitoring value. Peripheral inflammation (TNFα) may have added value along with clinical predictors of response.